# Abundant iron and sulfur oxidizers in the stratified sediment of a eutrophic freshwater reservoir with annual cyanobacterial blooms

**DOI:** 10.1038/srep43814

**Published:** 2017-03-07

**Authors:** Long Jin, Chang Soo Lee, Chi-Yong Ahn, Hyung-Gwan Lee, Sanghyup Lee, Hyeon Ho Shin, Dhongil Lim, Hee-Mock Oh

**Affiliations:** 1 College of Biology and the Environment, Co-Innovation Centre for Sustainable Forestry in Southern China, Nanjing Forestry University, Nanjing 210-037, China; 2 Culture Collection Team, Freshwater Bioresources Culture Research Division, Nakdonggang National Institute of Biological Resources, Sangju 37242, Republic of Korea; 3 Cell Factory Research Centre, Korea Research Institute of Bioscience & Biotechnology (KRIBB), Daejeon 34141, Republic of Korea; 4 Centre for Water Resource Cycle Research, Korea Institute of Science & Technology (KIST), Seoul 02792, Republic of Korea; 5 South Sea Research Institute, Korea Institute of Ocean Science & Technology (KIOST), Geoje 53201, Republic of Korea

## Abstract

The microbial community in eutrophic freshwater sediment was investigated from a 67-cm-deep sediment core collected from the Daechung Reservoir in South Korea, where cyanobacterial blooms have occurred annually for the past 30 years. The majority of core sediments were characterized by dark-grayish, fine-grained mud with abundant gas-escaped and thinly laminated layers. Intervals of summer and winter seasons were represented by periodic peaks of geochemical profiles of parameters such as grain size and relative carbon mass ratios to various nutrients such as nitrogen, sulfur, and phosphorus. In bacteria, *Proteobacteria* (66.6%) was the most prevalent phylum, followed by *Chloroflexi* (8.9%), *Bacteroidetes* (5.1%), and *Spirochaetes* (2.6%). Archaea were also abundant, representing approximately half of the total prokaryotes in the sediments. Notably, three *Bacteria (Sulfuricurvum, Sideroxydans*, and *Gallionella*) and one *Archaea (Thermoplasmata*) accounted for 43.4% and 38.4% of the total bacteria and archaea, respectively, implying that iron and sulfur oxidizing microorganisms dominate in this eutrophic freshwater sediment. These results indicate that 1) eutrophic freshwater lakes in monsoon climates undergo a stratified sedimentary process with seasonal and annual variations in geochemical and microbial profiles, and 2) the microbial oxidative metabolism of iron and sulfur is notably active in sediments from a eutrophic lake.

The eutrophication of freshwater ecosystems is an important environmental issue worldwide. Under a monsoon climate, artificial reservoirs may experience rapid eutrophication beyond natural levels because of the influx of abundant nutrients from the surrounding heavily populated watershed. Subsequently, the organic and inorganic nutrients stimulate the growth of microalgae and cyanobacteria, thus resulting in annual outbreaks of algal blooms in the water body and the formation of sediments on the bottom of the reservoir^1^.

Sediments play an intermediate role in the eutrophication of freshwaters and may result in the release or uptake of nutrients^2^. In regions with a monsoon climate, sediments may show seasonal or annual patterns depending on the type and amount of inflowing materials during the rainy season. The benthic ecosystem in freshwater may be greatly affected by the decomposition of organic compounds produced by photosynthetic organisms such as algae, cyanobacteria, and plants, especially during the rainy season^3^. The microbial use of inorganic nutrients is also an important factor in the management of the redox-dependent phosphorus cycle in eutrophic sediments^4^. For example, phosphorus-accumulating organisms accumulate phosphorus as poly-phosphate into biomass, which makes up at least 10% of the total phosphorus in sediments^5^. Moreover, the accumulated phosphorus in the sediments influences the aquatic microbial community, including cyanobacterial blooms the following summer^6^.

The eutrophication of freshwater ecosystems is also affected by the geochemical cycles of sediments^7^. Of the biological components of the phosphorus geochemical cycle, iron plays a critical role in the release and uptake of phosphorus through the reduction or oxidation of iron oxide. Thus, oxygenated sediments retain phosphorus by fixation into iron-phosphorus complexes; however, reduced sediments release phosphorus through the reduction of iron and the subsequent dissolution of the iron-phosphorus complexes. The roles of some iron-reducing bacteria have been highlighted for their dissimilatory capabilities in iron reduction during the formation of iron-phosphorus complexes^8^.

Microorganisms are regarded to be among the most important players in freshwater ecosystems by transforming or mineralizing organic matters. More specific functions of the freshwater microbes can be revealed using technologies such as deep sequencing. To date, many studies have examined the microbial community in the water column, whereas research on the microbial community of the sediments has been limited. The aim of this study was to identify the potential links between microbial profiles and geochemical cycles in the sediments of bloom-frequent freshwaters. Therefore, we investigated 1) the microbial community structure in the stratified sediments of a eutrophic freshwater reservoir and 2) the relationship between the microbial components and the environmental parameters. We surveyed the Daechung Reservoir as a model site for a freshwater reservoir, in which frequent annual cyanobacterial blooms have been observed particularly over the past 20, after the construction of the reservoir in 1980^9^,^10^,^11^.

## Results

### Lithology

**Figure 1:**
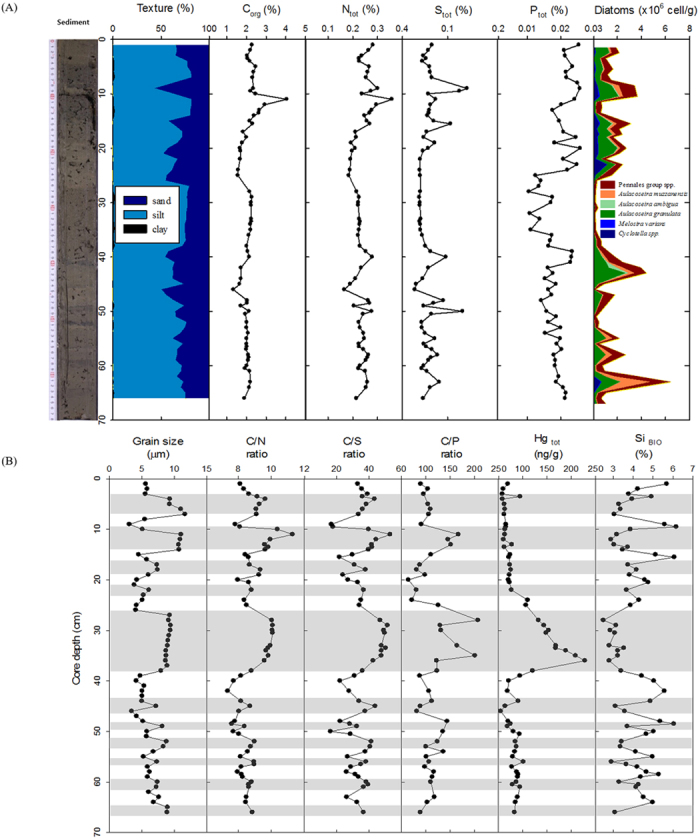
Geochemical depth profiles of sediments from the Daechung Reservoir. (**A**) Photograph of the Daechung Reservoir core sediments and down-core variations of sediment texture, element composition, and diatom assemblage as a function of core depth. (**B**) Seasonal variations in sediment grain size, the ratios between elemental composition, total mercury concentration (Hg_tot_) and biogenic silica content (Si_BIO_). Gray layers indicate the summer depositions with relatively coarser sediment grains, high C/N and C/S ratios, high soil-derived Hg level, and low abundance of Si_BIO_ and diatoms, but the winter depositions show the opposite.

Core sediments were primarily olive gray (5Y 4/2) and/or dark olive gray (5Y 3/2) with silt and clay particles and with abundant microbe-induced small gas escape holes (e.g., H_2_S and/or CH_4_) and faint partial laminations in the lower part of the core (Fig. 1A). Of particular note, several dark black (2.5/N) bands (particularly those with a 10–14 cm interval) and a thick, dark grayish brown (10YR 4/2) sediment layer intercalated between 28 and 38 cm were observed in the core. The sand grains were rare (<2%) throughout the core, and the silt and clay fractions ranged from 44% to 80% (average value of 68 ± 8%) and from 18% to 57% (average value of 31 ± 9%), respectively (Fig. 1A). The mean grain size of the core sediments varied from 3 to 12 microns, with an average grain size of 7.0 ± 2.2 microns (very fine silt); the grain size repeatedly switched between relatively clay-deficient silt with a mode grain size of 15.6 microns, including some sand particles, and clay-enriched mud with a mode grain size of 6 microns (Fig. 1B). The core showed systematic fluctuations in grain size in which the grain size showed high positive correlations with the C_org_ and C/N, C/S, and C/P ratios, whereas negative correlations were found with the Si_BIO_ content and diatoms such as *Aulacoseira granulata (p* < 0.001; Table 1).

### Carbon, nitrogen, sulfur and phosphorus profiles

The dark-colored bands and the brownish layer had a relatively high content of carbon, nitrogen, and sulfur with fluctuations within an order of magnitude. The total organic carbon (C_org_) and total nitrogen (N_tot_) content fluctuated from 1.33% to 4.04% (average value of 2.11 ± 0.37%) and from 0.17% to 0.36% (average value of 0.24 ± 0.03%), respectively (Fig. 1A). The correlation between C_org_ and N_tot_ was positive and significant (*r* = 0.835, *p* < 0.001; Table 1).

The total sulfur (S_tot_) content ranged from 0.04% to 0.14% (average value of 0.06 ± 0.02%) and fluctuated sharply, exhibiting alternating high and low peaks within the range, except at the middle of the core (Fig. 1A). The linear relationship between S_tot_ and N_tot_ was significant (*r* = 0.663, *p* < 0.001) (Table 1).

The level of total phosphorus (P_tot_) in the sediment samples fluctuated between 104 and 259 μg/g (average value of 191 ± 39 μg/g; Fig. 1A). The P_tot_ levels in the middle sediment layers (~20–40 cm) were relatively higher than those in the upper (~0–20 cm) and lower sediment layers (>40 cm). The C/P ratios ranged from 70 to 219, with an average value of 115 ± 32, and major peaks in the C/P ratios were found from sediment layers at depths of ~10–15 cm and ~25–35 cm (Fig. 1B). Additionally, the level of P_tot_ showed a positive relationship with both S_tot_ (*p* < 0.01) and N_tot_ (*p* < 0.05) but a negative relationship with Hg_tot_ (*p* < 0.05; Table 1).

### Biogenic silica, diatoms and mercury

The biogenic silica (Si_BIO_) content varied between 2.5% and 6.2% (average value of 4.1 ± 0.9%), and alternation of distinctly low and high Si_BIO_ contents occurred repeatedly throughout the entire core (Fig. 1B). Particularly, relatively low Si_BIO_ content was found in the brownish sediment intervals between 28 cm and 38 cm (Fig. 1A). The diatoms were primarily *Aulacoseira*, such as *A. granulate* and *A. muzzanensis. Cyclotella* and some Pennale group species, such as *Fragilaria crotonensis* and *Synedra ulna*, were also observed (Fig. S1). The diatoms showed positive correlations with S_tot_ and Si_BIO_, but negative correlations with the C/N and C/S ratios (Table 1).

The Hg_tot_ content ranged from 55.5 ng/g to 227.8 ng/g, with an average of 91.9 ± 37.6 ng/g, and the Hg_tot_ content dramatically increased in the brownish sediment layer (29–39 cm interval). With the exception of this interval, the Hg_tot_ content was largely maintained below 100 ng/g (Fig. 1B). The Hg_tot_ showed a high positive correlation with the C/N and C/S ratios but a negative correlation with S_tot_, Si_BIO_, P_tot_, and diatoms (Table 1).

### Bacteria

**Figure 2:**
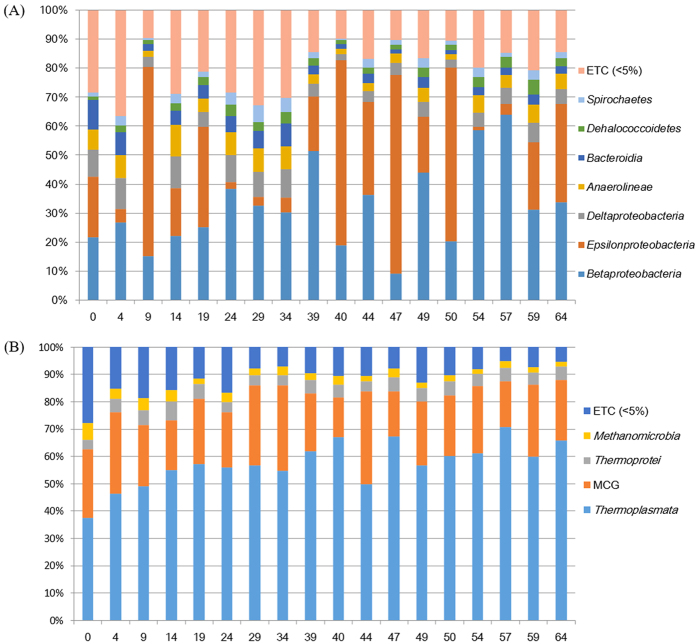
Taxonomic classification of bacterial (**A**) and archaeal (**B**) communities (class levels) in samples from each depth. Taxonomic classification of bacterial reads retrieved from pooled DNA amplicons from different seasonal water masses into phylum (**A**) and class (**B**) levels using the RDP classifier. The names for each color appear below the figure. The nomenclature for each phylotype is based on the EzTaxon-e database.

*Proteobacteria* (66.6%) was the most prevalent phylum in all sediment samples, followed by *Chloroflexi* (8.9%), *Bacteroidetes* (5.1%), *Spirochaetes* (2.6%), *Planctomycetes* (2.4%), and *Acidobacteria* (2.2%). The other divisions were present only as minor components. The most abundant classes in the sediment libraries were *Betaproteobacteria* (32.2%) and *Epsilonproteobacteria* (26.5%) (Fig. 2A). The three most abundant genera were *Sulfuricurvum, Sideroxydans*, and *Gallionella*, which represented 26.3%, 13.0%, and 5.2%–80% of the total bacteria, respectively (Fig. S3).

However, important differences in the relative distribution of the subdivisions were observed. *Betaproteobacteria* was predominant in all sediment samples up to 64.0%. *Epsilonproteobacteria* was also predominant up to 68.5%, with the exception of samples at depths of 4, 29, 34, 54, and 57 cm. Compared with *Betaproteobacteria* and *Epsilonproteobacteria,* the *Alpha*-, *Gamma*- and *Delta*- subdivisions represented minor components in the sediment samples (Fig. S2). The genera *Gallionella* and *Sideroxydans* were dominant in *Betaproteobacteria*, while the genus *Sulfuricurvum* was the most representative in *Epsilonproteobacteria*. Three genera, *Sulfuricurvum, Sideroxydans*, and *Gallionella*, represented approximately 50% of the bacteria in the sediment samples (Fig. S3).

### Archaea

Various class-level taxa of archaea were identified in the core of the sediment samples, but their relative abundance varied greatly (Fig. 2B). *Thermoplasmata* was the most abundant archaeal class in all sediment samples. *Thermoplasmata* contributed ≥60% of the total number of small subunit (SSU) rRNA gene sequence reads in six samples (39, 40, 47, 50, 54, 57, and 64 cm; Fig. S4). The miscellaneous *Crenarchaeota* group (MCG) was the second most abundant group of archaea in all sediment samples and contributed 14–31% of the total archaeal SSU rRNA gene sequence reads. Notably, the relative abundance of MCG in all samples correlated significantly with both the C/N and C/S ratios (Fig. S5). Compared with *Thermoplasmata* and the MCG, methanogens, such as *Methanomicrobia* (2.9%) and *Methanobacteria* (0.4%), were identified as minor components of the total archaea.

### Microbial abundance

Quantitative PCR was used to estimate the number of SSU rRNA genes in the sediment samples to detect archaea, bacteria, cyanobacteria, and plastid in the photosynthetic organisms. The total number of SSU rRNA genes in the core (*Archaea* plus *Bacteria*) varied between 2.4 × 10^8^ and 5.1 × 10^8^ 16S rRNA gene copies/g of sediment (wet weight). The archaeal 16S rRNA gene copy number contributed 25.1*–*57.8% of the total number of SSU rRNA gene copies. The abundance of cyanobacterial 16S rRNA and the plastid 23S rRNA gene markers were in the range of 10^4^ to 10^7^. In the cyanobacterial 16S rRNA and the plastid 23S rRNA gene markers, the highest numbers were found in the top layers, whereas the lowest levels were observed in the bottom layers (Table S2).

### Statistical analyses

**Figure 3:**
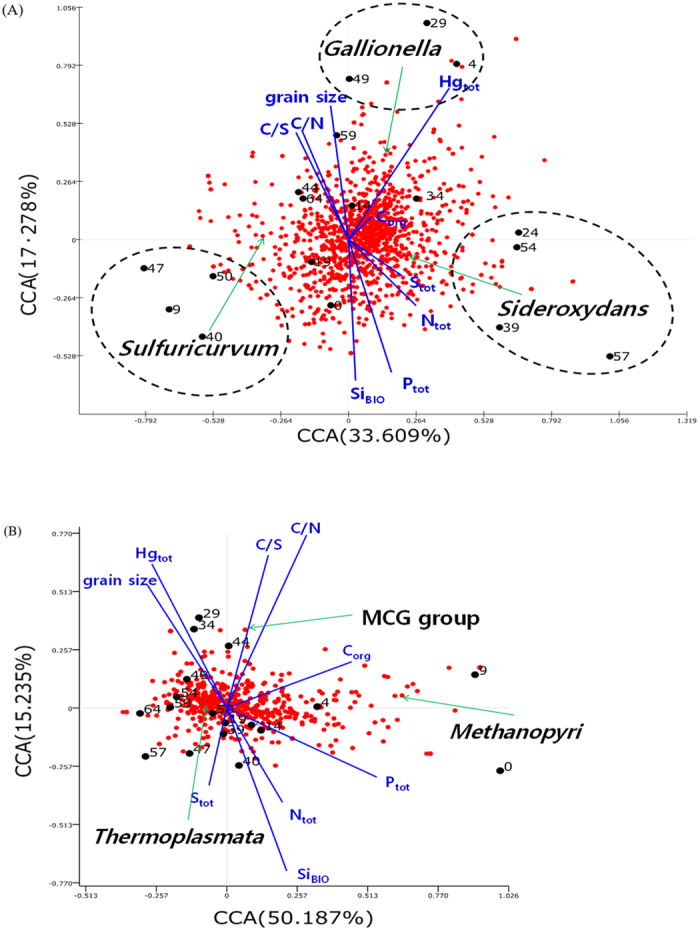
Canonical correlation analysis (CCA) plots to determine the relationships among the abundance of bacteria (**A**) and archaea (**B**) and the geochemical factors of the samples. The percentages on each axis represent the variation in samples. The circles indicate the samples, and the red circles indicate the genera of bacteria.

Correlations between the geochemical factors and the microbial communities in all samples were analyzed by canonical correlation analysis (CCA). In contrast to the Hg_tot_ concentrations, samples with depths of 9, 40, 47, and 50 cm formed an isolated group (Fig. 3A). One common attribute of this cluster was the high proportion (59.5% to 68.2%) of *Sulfuricurvum* (Fig. S3). The location of *Sideroxydans* in the CCA plot was more closely related with samples from depths of 24, 39, 54, and 57 cm, as these depths were characterized by a higher proportion of *Sideroxydans* (23.2% to 56.9%) than other depths (Fig. 3A). Although both *Sideroxydans* and *Gallionella* are iron-oxidizers in the family *Gallionellaceae*, the locations of the two genera in the sediments were sharply differentiated (Fig. 3A). The *Gallionella* point was located near samples from depths of 4, 29, and 49 cm in the first quadrant of the CCA plot, where the proportion of *Gallionella* increased to over 10% (Fig. S6).

## Discussion

### Seasonal variations in lake sedimentation

The grain sizes and geochemical compositions in the core, including the ratios between the two, showed distinct high-amplitude fluctuations with periodicities of approximately 2–10 cm intervals; furthermore, such variations were all connected systematically (Fig. 1). The repetitive grain size fluctuations, with alternate layers of fine silt in the mean grain size, most likely reflected the seasonal changes that occurred in lake sedimentation. An increase in lake sediment grain size may be indicative of many phenomena such as an increase in runoff coupled with strong streams and river flow into the lake in the East Asian monsoonal climatic area^12^. During summer floods, the high rates of precipitation may increase the soil erosion of the surrounding area and the transport capacity of streams and rivers, thus leading to subsequent depositions of coarser clastic materials in the lake^13^. Accordingly, we inferred that the fluctuations in the relatively coarse grain size layers (primarily >7 microns) with some sand grains (Fig. 3) were predominantly related to depositions that accumulated during the summer monsoon season, i.e., the grain size fluctuations in the core yielded the pattern of coarser sediments during the summer periods and the pattern of finer sediments during the dry winter periods.

These season-dependent variations in the sizes of sediment particles were closely linked with large fluctuations in the geochemical components, particularly the C/N ratio and Si_BIO_ and Hg_tot_ contents (Fig. 1). Because terrestrial plant C/N ratios are higher (>20) than those of the aquatic plankton (<10), owing to more cellulose in the terrestrial organic matter and greater amounts of nitrogen in the phytoplankton^14^, variations in the C/N ratio can provide a measure of the amount of terrestrial versus aquatic organic inputs into the lake sediments. In lake core sediments from the study area, the C/N ratios ranged from 7 to 11 (average value of 9 ± 1), which are typical values for lakes, in which the organic materials are primarily derived from freshwater planktonic organisms^15^. Therefore, the significant increase in the C/N ratios of the core was most probably caused by an increase in the proportion of terrestrial organic matter in lake sediments. Notably, the variations in C/N ratios coincided exactly with the seasonality of grain size (Fig. 1A). Thus, the C/N ratios were relatively high in the coarse-grained summer sedimentation layers, thus suggesting a relative increase in the amount of land-based terrestrial organic matter input from soil materials with high C/N ratios that entered the lake during the summer season as a result of high rates of precipitation. This relationship was apparent in the brownish sediment layer in the high Hg_tot_ content that was temporarily supplied from the topsoil by a catastrophic flooding event. The clearly delineated stepwise layers of increasing Hg_tot_ content may be associated with heavy rainfall or strong flooding near the surrounding areas and the subsequent sedimentation of eroded surface soil into the lake^16^,^17^.

The C/S ratio was relatively low in the fine-grained winter sediment layers, but was much higher in the coarse-grained summer sediment layers (Fig. 1B). The C/S ratios fluctuated substantially within a range of 17–53 (average value of 36 ± 9) throughout the entire core, and these values were significantly lower than those of average lake sediments (~80)^18^. These low ratios, combined with the gas-exposed structures, suggest a high chance of sulfate reduction and pyrite formation in the anoxic sediment layers^19^,^20^. In addition, the positive correlation between the S_tot_ and the P_tot_ content (*p* < 0.01) also correspond with simultaneous sulfate reduction and pyrite formation. In contrast, the high C/S ratios maybe attributed to the elevated carbon influx into the sediment by increased photosynthetic productivity (i.e., high chlorophyll *a*) and its consequent high rate of organic matter sedimentation during the summer^21^. Lake basins are relatively rich in reduced sulfur during the winter deposition, but the conditions may be opposite during the summer season^22^. A higher O_2_ concentration in the water body during the summer season prevents the accumulation of sulfide released from the sediment because the sulfide is rapidly oxidized^23^. Indeed, the dissolved oxygen and chlorophyll concentrations at the study site were much higher in the summer season than in the winter season (Fig. S7).

### Diatoms

Prominent variations in diatom abundance and Si_BIO_ content facilitated our interpretation of the seasonal records in the sediment. In general, the density of diatoms reaches a maximum during the late fall or winter, whereas the lowest density occurs during the summer in monsoonal climatic areas^24^,^25^,^26^. In this study, the variations in diatom abundance were consistent with the season-dependent variations observed in grain size and geochemical components. In the core sediments, the high peaks in diatom abundance clearly corresponded to the intervals of the winter seasons, and the low peaks clearly corresponded to the summer season intervals. The diatoms were rare, particularly in the thick, brownish coarse-grained sediment interval with high C/N and C/S ratios, which indicated a strong summer monsoonal flood event.

Additionally, *Aulacoseira granulata*, which is known to be most abundant during the late fall and/or winter seasons with low water temperatures in the lake^25^,^26^, was the most dominant species in the diatom assemblages of the core (Fig. S1). This result indicated that the diatom blooms in the study lake occurred during the cold seasons. Notably, the *Cyclotella* species were abundant in the upper part of the core, particularly after a strong summer monsoonal event; *Cyclotella* species are more commonly observed at a higher abundance in lakes with low nutrients (i.e., oligotrophic) than in lakes that are eutrophic^27^,^28^.

### Bacteria

Most members of the bacterial community were closely related to cultivated sulfur-oxidizing species or species from environments such as deep-sea vents, caves and groundwater. These members are likely to be involved in sulfur cycling, including the oxidation of sulfide by using small amount of oxygen or nitrate. A variety of neutrophilic and acidophilic iron-oxidizing microorganisms derive energy for growth from the oxidation of dissolved or structural Fe(II) under either oxic or anoxic conditions. Recent studies show that aerobic neutrophilic iron-oxidizing bacteria would play an important role in microoxic niches with low O_2_. Anaerobic ferrous iron oxidizing microorganisms oxidize ferrous oxide by using nitrate under anaerobic conditions at a neutral pH, thus driving active microbial anoxic redox cycling of iron^29^.

The genus-level analyses of the sediment bacteria revealed considerable divergence in the abundance of the major genus-level taxonomic groups. In most samples, the three most abundant genera, *Sulfuricurvum, Sideroxydans*, and *Gallionella*, represented approximately 50% of the total bacteria (Fig. S3). *Sulfuricurvum* spp. are sulfur-oxidizing bacteria that use sulfide, elemental sulfur, thiosulfate and hydrogen as electron donors and nitrate as the electron acceptor under anaerobic conditions^30^. *Gallionella* spp. are iron-oxidizing chemolithotrophic bacteria that live under low-oxygen conditions^31^. *Gallionella ferruginea* oxidizes dissolved iron, thereby removing this iron from the water and producing an insoluble precipitate of ferric hydroxide. *Sideroxydans* spp. grow on FeCO_3_ or FeS at oxic-anoxic interfaces at a circumneutral pH. However, the molecular mechanisms of oxidizing Fe(II) remain unknown.

Iron oxidation by *Sideroxydans* and *Gallionella* in the sediments is an important metabolic function of *Sideroxydans* spp. and *Gallionella* spp.^32^. Despite their similar chemolithoautotrophy (i.e., iron oxidation), *Sideroxydans* and *Gallionella* showed different abundance patterns in the sediments (Fig. S3). A previous comparative genomic analysis has reported that both genera use metabolic pathways such as carbon dioxide fixation and ferrous oxide oxidation^33^. *Sideroxydans* is more metabolically flexible and uses reduced sulfur compounds, whereas *Gallionella* contains additional gene clusters for exopolysaccharide production and exhibits acid and metal tolerance^33^,^34^. Thus, the differentiated adaptations to various environments may have an effect on their separate ecological niches.

### Archaea

The most abundant archaeal group was *Thermoplasmata*, which are typically acidophilic, aerobic, mesophilic to thermophilic. Additionally, environmental clones were found in ordinary environments. Many species within the class *Thermoplasmata* that play important roles in the iron and sulfur cycles were identified^35^. The abundance of MCG in the sediment samples and the correlations between MCG with both the C/N and the C/S ratios may be because the heterotrophy of MCG was coupled with the reduction of sulfur compounds. This MCG group has been proposed to be a group of anaerobic heterotrophs that have no cultured representatives^36^. Additionally, the archaeal community contained a methanogen archaeon, *Methanopyri*, in the top layers of the sediment core from a depth of 0 to 9 cm; carbon, nitrogen, and phosphorus were also abundant in the shallow sediments. The distribution patterns of archaea and bacteria are indicated by red dots in the CCA plot (Fig. 3). Compared with the well-distributed pattern of bacteria (Fig. 3A), archaeal members were partially biased on the right side on the x-axis (Fig. 3B). When compared with environmental factors, the CCA ordination analysis indicated that organic carbon (C_org_) and total phosphorus (P_tot_) were important variables that influence the archaeal community structure including *Methanopyri* in the sediment.

### Process of freshwater sedimentation

Although the relationships between nutrients and microbial communities are highly complex, it was apparent that the microbial community structure was strongly correlated with the iron and sulfur content. We examined the microbial mineralization in the iron and sulfur cycles in freshwater sediments. For example, the sulfur-reducing bacteria were less abundant than the sulfur-oxidizing bacteria in this study, a result that may have been caused by the limited sulfate concentrations in the freshwater sediments compared with those in marine systems and the subsequent limited activity of sulfate-reducing bacteria^37^. In the present study, the disproportionation of elemental sulfur coupled to Fe reduction compensated for the reducing power in the freshwater sediments by anaerobic sulfide oxidation under limited sulfate-reducing activity^38^. A neutrophilic iron-oxidizing OTU has been found under nitrate-reducing condition and is most closely related to *Gallionella* on the basis of 16S rRNA similarity^39^. These previous results suggest that the relationship between sulfur oxidizers and iron oxidizers provides a syntrophic interaction between ferrous oxide oxidation and dissimilatory ferric oxide reduction^40^.

**Figure 4:**
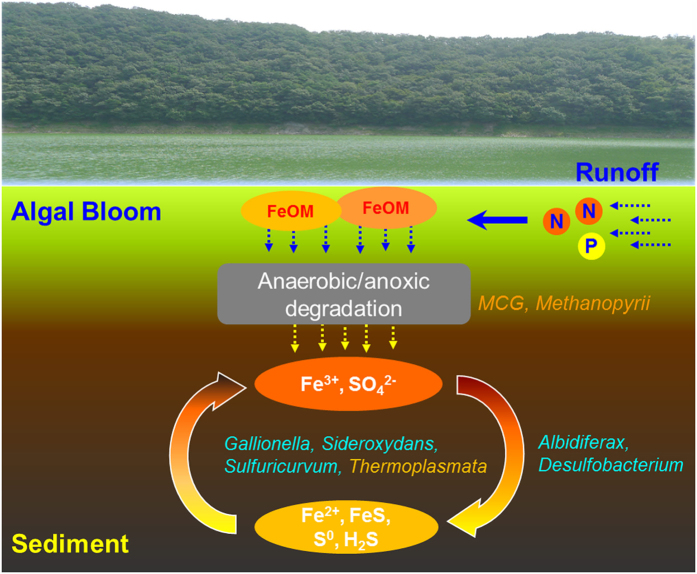
Biogeochemical cycles of organic and inorganic nutrients in the sediments from the Daechung Reservoir. Organic and inorganic nutrients can flow into freshwater by heavy rainfall in monsoon climates, followed by the algal growth and its precipitation with iron. In the top sediment, the organic matter with iron (FeOM) is degraded by heterotrophic Bacteria and Archaea (MCG group), followed by the mineralization of iron and sulfur by abundant iron and sulfur oxidizers (*Gallionella, Sideroxydans, Sulfuricurvum*, and *Thermoplasmata*). The reducing powers of iron and sulfur oxidation can also be obtained from iron-reducing bacteria (*Albidiferax*) and sulfur-reducing bacteria (*Desulfobacterium*).

Under anoxic conditions driven by the degradation of organic carbon, abundant iron and sulfur can be used by iron and sulfur oxidizers^41^. Therefore, microorganisms play important roles throughout the entire process of sedimentation and eutrophication in a freshwater reservoir. The process of freshwater sedimentation has been hypothesized to occur as a result of the influx of organic and inorganic nutrients. The organic carbon deposition with iron occurs with the growth of algae and cyanobacteria, followed by the precipitation and subsequent degradation of organic carbon by heterotrophic bacteria and archaea (e.g. MCG group). Finally, the sedimentation process leads to the mineralization of inorganic nutrients such as iron and sulfur by abundant iron and sulfur oxidizers. In addition, iron-reducing bacteria such as *Albidiferax* and sulfur-reducing bacteria such as *Desulfobacterium* were also observed (data not shown), thus suggesting that the reducing powers of iron and sulfur oxidation can also be closely coupled by microbial components in the freshwater sediments (Fig. 4)^42^. Consequently, in eutrophic freshwater lakes in monsoon climates, a stratified sedimentary process with seasonal and annual variations in the geochemical and microbial profiles was observed. This observation may be closely related to the precipitation and subsequent biodegradation of organic carbon derived from the growth of algae and cyanobacteria. In particular, the microbial oxidation of iron and sulfur in the sediments are notably active.

## Materials and Methods

### Site location and sediment sampling

The Daechung Reservoir is located at the upper part of the Geum River in the central region of South Korea. This reservoir is a large branch-type lake with a 72-m-high dam and a gross storage capacity of 1,490 Mm^3^. The reservoir has received primarily agricultural runoff and becomes a representative cultural eutrophic lake after its construction in 1980. The sampling site was located on the shore in the vicinity of the Daechung Reservoir dam^9^. A 67-cm-long sediment core was collected from the Chusori of the Daechung Reservoir at a 17-m water depth using a modified gravity corer (36°22′30″ N, 127°33′58″ E).

### Physicochemical analyses

The core was split longitudinally and photographed, and the details were logged on the basis of visual examination. Subsamples were collected from the core at 0.5–2 cm (primarily 1 cm) intervals to determine grain size, nutrient and total Hg contents, as well as the content of biogenic silica and diatom assemblages. The dry weight of the sediments was determined using a drying chamber (Convection oven MOV-112F; Sanyo Electric, Tokyo, Japan) at 105 °C overnight, and the analysis was accurate within a 5% relative error. The grain size of the sediments was analyzed using a laser diffraction particle size analyzer (HELOS/RODOS&SUCELL, Sympatec GmbH, Germany) after the removal of organic matter and carbonates.

The total nitrogen (N_tot_), carbon (C_tot_) and sulfur (S_tot_) content was measured using a Carlo Erba elemental analyzer 1108 (CE Instruments, Milan, Italy). The total inorganic carbon (C_inorg_) content was measured using an UIC CO_2_ coulometer (model CM5014). The total phosphorus (P_tot_) was analyzed by the acid persulfate digestion method according to standard methods, using TP-LR and TP-HR kits (C-Mac, Daejeon, South Korea)^43^. The total Hg (Hg_tot_) content was determined using an analyzer with a CVAAS module (Hydra-C; detection limit, 0.005 ng Hg; Teledyne Leeman Labs, Hudson, NH, USA) based on the USEPA method 7473. The errors of accuracy and precision were determined by repeated analysis of standard reference materials (MESS-3, n = 7), together with a batch of sediment samples that were between 5% and 10%, which indicated satisfactory data acquisition. The biogenic silica (Si_BIO_) content was analyzed using a modified wet alkaline extraction method^44^,^45^. The relative error of Si_BIO_ content in the sediment samples was less than 3%.

To determine diatom abundance and species composition, the sediment samples were treated according to a modification of the panning method^46^. Approximately 1.0 g wet weight of each sample was resuspended in 10 ml sterilized water and sonicated twice for 5 seconds (UT-53 N; Sharp, Japan) to separate the cells from the sediment. The suspension was size-fractionated with 120 μm and 10 μm nylon mesh screens and concentrated to a final sample volume of 10 ml; samples were then stored in the dark at 4 °C. For the enumeration of diatoms, 100 μl of each sample was placed in a Sedgwick-Rafter counting chamber, and the density of intact cells was counted using an upright microscope (Zeiss, Germany) at x200 magnification. The counts were converted into number per gram of wet weight sediment. In this study, the abundance is shown as the sum of living and empty cells.

### Sequencing and quantitative PCR

Total DNA was extracted from sediment samples (500 mg) using a FastDNA SPIN Kit for Soil and FastPrep Instruments (MP Biomedicals, Santa Ana, CA, USA). After verification of the quantification of the extracted DNA on an ND1000 spectrometer (NanoDrop Technologies Inc., Wilmington, DE, USA), the 16S rRNA gene fragments that contained the V1–V3 regions were amplified by PCR using a C1000 Touch Thermal Cycler (BioRad, Hercules, CA, USA) as previously described^47^. Subsequent bar-coded PCR amplification, sequencing, and pipeline processes were conducted by ChunLab, Inc. (http://www.chunlab.com), thus yielding a 16S rRNA gene amplicon library with a total of 313, 540 high-quality sequence reads for sediment samples. All pyrosequencing reads obtained in this study have been submitted to the Sequence Read Archive (SRA) of NCBI under study accession number SAMN03788027-SAMN03788028. A detailed pyrosequencing protocol is described in the Supporting Information.

Four qPCR assays were used to quantify the plastid rRNA gene of photosynthetic microorganisms, the cyanobacterial 16S rRNA gene, the bacterial 16S rRNA gene, and the archaeal 16S rRNA gene. Each genetic marker was amplified with a primer set: p23SrV_f1 (5′-GGA CAG AAA GAC CCT ATG AA-3′) and p23SrV_r1 (5′-TCA GCC TGT TAT CCC TAG AG-3′) for the plastid rRNA gene^48^; CYA359F (5′-GGG GAA TYT TCC GCA ATG GG-3′) and CYA781R (5′-GAC TAC WGG GGT ATC TAA TCC CWT T-3′) for the cyanobacterial 16S rRNA gene^49^; Uni331F (5′-TCC TAC GGG AGG CAG T-3′) and Uni797R (5′-GGA CTA CCA GGG TAT CTA ATC CTG TT-3′) for the bacterial 16S rRNA gene^50^; and Uni331F (5′-TCC TAC GGG AGG CAG T-3′) and modified Arc908R (5′-CCC GCC AAT TCC TTT AAG TT-3′) for the archaeal 16S rRNA gene^51^. A detailed qPCR protocol and a quantification procedure are described in the Supporting Information.

### Statistical analyses

Canonical correlation analysis (CCA) was performed to analyze the relationships among the physicochemical factors and the microbial community by using the R statistical software package (ver 2.15.2)^52^. All species in each sample were used as the community matrix for the CCA based on the chi-squared distance, and the significance of a correlation was assessed with a 1,000 permutations test using a mock ANOVA. The linear fitting function (“env_fit”) was used to explain the significant environmental components in relation to the cyanobacterial genus^53^. A stress value of 10 was considered to be an indicator of good ordination, and the significance of each vector was assessed with a goodness-of-fit statistic (*r*^*2*^) using 1,000 permutations. The environmental factors that correlated significantly (*p* < 0.05) were selected and are presented in the plots^54^.

## Additional Information

**How to cite this article**: Jin, L. *et al*. Abundant iron and sulfur oxidizers in the stratified sediment of a eutrophic freshwater reservoir with annual cyanobacterial blooms. *Sci. Rep.* **7**, 43814; doi: 10.1038/srep43814 (2017).

**Publisher's note:** Springer Nature remains neutral with regard to jurisdictional claims in published maps and institutional affiliations.

## Supplementary Material

Supplementary Information

## Figures and Tables

**Table 1:** Pearson correlation analysis among biological, chemical, and physical factors in the sediments (*n* = 55).

	Mean grain size	N_tot_ (%)	C_org_ (%)	S_tot_ (%)	C/N	C/S	Si_BIO_ (%)	Hg_tot_ (ng/g)	P_tot_ (μg/g)	C/P	*Aulacoseiragranulata*	Pennales	Total diatoms
Mean grain size	1.000												
N_tot_ (%)	0.211	1.000											
C_org_ (%)	0.558**	0.835**	1.000										
S_tot_ (%)	−0.287	0.663**	0.294	1.000									
C/N	0.732**	0.084	0.609**	−0.384*	1.000								
C/S	0.657**	−0.147	0.341*	−0.740**	0.823**	1.000							
Si_BIO_ (%)	−0.727**	0.257	−0.198	0.628**	−0.731**	−0.797**	1.000						
Hg_tot_ (ng/g)	0.282	−0.232	0.000	−0.337*	0.401*	0.475**	−0.414*	1.000					
P_tot_ (μg/g)	−0.070	0.347*	0.230	0.265	−0.116	−0.213	0.250	−0.513**	1.000				
C/P	0.494**	0.300	0.505**	−0.006	0.573**	0.453*	−0.373*	0.499**	−0.678**	1.000			
*Aulacoseira granulata*	−0.463**	0.080	−0.142	0.457*	−0.403*	−0.640**	0.637**	−0.445*	0.414*	−0.451*	1.000		
Pennales	−0.173	0.277	0.036	0.561**	−0.330*	−0.579**	0.492**	−0.391*	0.107	−0.175	0.727**	1.000	
Total diatoms	−0.305	0.163	−0.062	0.523**	−0.362*	−0.625**	0.572**	−0.385*	0.235	−0.288	0.902**	0.907**	1.000

^*^p < 0.05 and **p < 0.001.
