# Supplementary material for: Abundant iron and sulfur oxidizers in the stratified sediment of a eutrophic freshwater reservoir with annual cyanobacterial blooms
Source: Sci Rep. 2017 Mar 7;7:43814. doi: 10.1038/srep43814 (PMC5339789; doi:10.1038/srep43814)
Supplement: Supplementary Information [file srep43814-s1.pdf]

Abundant iron and sulfur oxidizers in the stratified sediment of a eutrophic freshwater reservoir with annual cyanobacterial blooms

Long Jin[1,*], Chang Soo Lee[2,*], Chi-Yong Ahn[3], Hyung-Gwan Lee[3], Sanghyup Lee[4], Hyeon Ho Shin[5], Dhongil Lim[5,#], and Hee-Mock Oh[3,#]

[1] College of Biology and the Environment, Co-Innovation Centre for Sustainable Forestry in Southern China, Nanjing Forestry University, Nanjing 210-037, China

[2] Culture Collection Team, Freshwater Bioresources Culture Research Division, Nakdonggang National Institute of Biological Resources, Sangju 37242, Republic of Korea

[3] Cell Factory Research Centre, Korea Research Institute of Bioscience & Biotechnology (KRIBB), Daejeon 34141, Republic of Korea

[4] Centre for Water Resource Cycle Research, Korea Institute of Science & Technology (KIST), Seoul 02792, Republic of Korea

[5] South Sea Research Institute, Korea Institute of Ocean Science & Technology (KIOST), Geoje 53201, Republic of Korea

*: L. Jin and C. S. Lee contributed equally to this work.

[#] **Corresponding authors:**

Please contact Hee-Mock Oh, Tel.: +82-42-860-4321, Fax: +82-42-860-4594, e-mail: heemock@kribb.re.kr; or Dhongil Lim, Tel.: +82-55-639-8580, Fax: +82-55-639-8429, e-mail: oceanlim@kiost.ac.kr

Supplementary Materials

Pyrosequencing. For the PCR amplification, a bar-coded 16S rDNA tag approach was conducted with a bar-coded fusion primer at a concentration of 10 mM and 2 U of Taq polymerase (Ex Taq; Takara). The primer sequences were as follow: forward primer 5′-GCCTCCCTCGCGCCATCAGAGAGTTTGATYMTGGCTCAG-3′ and reverse primer 5′-GCCTTGCCAGCCCGCTCAGTIACCGIIICTICTGGCAC-3′. Unique bar codes were attached to the reverse primers of each sample, which were followed by a 2-base linker for the fusion primers (1). The amplified products were purified using the QIAquick PCR Purification Kit (Qiagen), and the quantification was conducted with a PicoGreen dsDNA Assay Kit (Invitrogen, Carlsbad, CA, USA). The equimolar concentrations of each amplified product were mixed and were purified again using an AMPure Bead Kit (Agencourt Bioscience, Beverly, MA, USA). The beads that were recovered after emulsion PCR were deposited on a 454-picotiter plate, and the sequencing was performed using a Roche/454 GS Junior system according to the manufacturer's instructions.

The raw sequence files were processed by (I) demultiplexing, (II) trimming the primer sequence, (III) quality filtering, (IV) sequencing error correction, (V) taxonomic assignment, and (VI) detection of chimeras. Each sample was identified by a unique bar code in the demultiplexing step, and the low quality reads (average quality score < 25 or read length < 300 bp) were removed from further analysis. The pairwise sequence alignment and the hmm-search program of the HMMER 3.0 package were used to trim the primer sequences based on the profile of the 16S rRNA V1–V3 regions (2). To correct the sequencing errors, representative sequences in the clusters of trimmed sequences were chosen for the taxonomic identifications. The individual reads were assigned to taxonomic positions according to the highest pairwise similarity among the top five BLASTN hits against the EzTaxon-e database

(3). The chimera sequences were removed by UCHIME (4). The read number in each sample was normalized with random subsampling. The species and the proportions of bacterial species that were shared between two samples or sets of multiple samples were calculated using the CLcommunity software (ChunLab, Inc., South Korea). The similarity coefficients for Bray-Curtis, Jaccard and Sorenson abundance were calculated using Mothur (5), and the matrix of Fast UniFrac was generated (6).

Quantitative PCR. Each qPCR mixture had a total volume of 25 µl that contained 5 µl of the 5-fold diluted DNA template, 12.5 µl of a commercially available master mix (RealHelix™ qRT-PCR kit; Nanohelix Co., Daejeon, South Korea), and 400 nM of each primer. The PCR cycling conditions included a common amplification step with an initial cycle at 50 °C for 2 min and at 95 °C for 10 min to activate the DNA polymerase, followed by 40 cycles of denaturation at 95 °C for 15 s, annealing at 60 °C for 30 s, and an extension at 72 °C for 30 s. To verify the specificity of the qPCR assays, each melting point analysis was conducted by increasing the temperature from 50 °C to 95 °C by 0.1 °C/s while measuring the fluorescence continuously. For the positive controls, *Chlorella vulgaris* KCTC 1006, *Microcystis aeruginosa* KCTC AG20763, *Methanobrevibacter boviskoreani* KCTC 4102, and *Bacillus subtilis* supsp. *subtilis* ATCC 6051 were obtained from the Korean Collection for Type Culture (http://kctc.kribb.re.kr) and were used for the plastid rRNA, the cyanobacterial 16S rRNA, the archaeal and the bacterial 16S rRNA gene markers, respectively. DNase/RNase-free water was used as a negative control. All measurements were conducted in duplicate on the Chromo4™ real-time PCR detector (BioRad, Hercules, CA, USA).

For the quantification of the markers, the PCR products were amplified identically as the qPCR assays with the template DNAs extracted from the positive strains. After

amplification with the Taq DNA polymerase kit (Invitrogen, Carlsbad, CA, USA) and purification with the QIAquick PCR Purification Kit (Qiagen, Valencia, CA, USA), the PCR products were cloned using the pGEM-T Vector System (Promega Co., Madison, WI, USA), which was followed by the blue-white screening and subculturing on Lysogeny Broth (LB) agar (BD Diagnostics, Sparks, MD, USA), according to the manufacturer's instruction. The plasmid DNAs of the subcultured clones were then extracted using the QIAprep Spin Miniprep Kit (Qiagen, Valencia, CA, USA), and the concentrations were measured with the NanoDrop spectrophotometer. Each 10-fold serial dilution was prepared using linearized plasmid DNA and was followed by each qPCR assay in duplicate with the Chromo4^{TM} real-time PCR detector (BioRad, Hercules, CA, USA) to construct each standard curve with the plotting of CT values versus log10 values of the gene copy number for which a limit of quantification (LOQ) was defined as the lowest gene copy number within the linear range of quantification.

Supplementary Table S1. Context data for sediment core sample. All gene copy numbers are per gram of wet weight sediment (-, missing value).

										Gene copies/g sediment				Cell number
Depth (cmbsf)	TP (mg/g)	TN (%)	TOC (%)	S (%)	C/N	C/S	B-Si (%)	Hg (ng/g)	Grain size	Bacteria (SSU)	Archaea (SSU)	Cyanobacteria (SSU)	Plastid (SSU)	Diatom (cell count)
0	0.026	0.281	2.284	0.069	8.1	33.2	5.7	69.9	5.6	3.7×10^{8}	1.4×10^{8}	5.2×10^{7}	5.8×10^{7}	1.9×10^{6}
4	0.023	0.266	2.468	0.064	9.3	38.4	3.3	62.7	9.3	2.4×10^{8}	1.5×10^{8}	5.2×10^{7}	3.7×10^{7}	1.4×10^{6}
9	0.028	0.237	2.463	0.062	10.4	39.8	3.9	64.1	5.1	1.1×10^{8}	1.4×10^{8}	9.1×10^{6}	4.2×10^{6}	3.6×10^{6}
14	0.017	0.257	2.165	0.072	8.4	29.9	5.1	74.8	4.5	1.9×10^{8}	1.3×10^{8}	1.5×10^{6}	1.3×10^{6}	1.9×10^{6}
19	0.025	0.210	1.670	0.062	8.0	27.2	4.6	71.2	4.2	1.4×10^{8}	1.4×10^{8}	4.4×10^{5}	5.1×10^{5}	2.2×10^{6}
24	0.026	0.184	1.564	0.046	8.5	34.1	3.9	107.0	4.2	2.3×10^{8}	1.0×10^{8}	3.4×10^{5}	3.1×10^{5}	2.0×10^{6}
29	0.018	0.222	2.237	0.045	10.1	49.2	2.8	153.5	9.4	1.2×10^{8}	1.5×10^{8}	8.8×10^{4}	1.3×10^{5}	2.1×10^{6}
34	0.013	0.225	2.213	0.046	9.8	47.7	3.2	208.8	8.7	1.4×10^{8}	9.9×10^{7}	2.5×10^{5}	3.1×10^{5}	2.2×10^{6}
39	0.021	0.278	2.149	0.097	7.7	22.2	5.0	71.8	4.1	3.8×10^{8}	1.3×10^{8}	8.4×10^{5}	6.0×10^{5}	1.3×10^{6}
40	0.026	0.234	1.722	0.062	7.4	27.8	5.6	69.3	5.0	2.2×10^{8}	1.1×10^{8}	5.6×10^{5}	1.2×10^{6}	2.5×10^{6}
44	0.019	0.190	1.654	0.038	8.7	43.9	3.1	64.4	7.2	1.5×10^{8}	1.5×10^{8}	9.5×10^{5}	6.3×10^{5}	2.7×10^{6}
47	0.012	0.262	2.039	0.092	7.8	22.3	5.4	70.6	5.2	1.7×10^{8}	1.3×10^{8}	2.0×10^{5}	1.8×10^{5}	1.8×10^{6}
49	0.018	0.277	2.131	0.129	7.7	16.5	5.0	81.1	5.8	1.8×10^{8}	1.1×10^{8}	8.2×10^{4}	1.2×10^{5}	-
50	0.016	0.225	2.018	0.049	9.0	41.5	3.4	85.4	8.8	2.1×10^{8}	1.5×10^{8}	8.2×10^{4}	1.5×10^{5}	2.8×10^{6}
54	0.021	0.246	2.006	0.075	8.1	26.9	5.0	80.0	5.3	2.9×10^{8}	2.0×10^{8}	7.6×10^{4}	2.0×10^{5}	1.3×10^{6}
57	0.023	0.263	2.094	0.080	8.0	26.2	4.7	88.3	6.2	2.2×10^{8}	1.9×10^{8}	4.7×10^{5}	4.9×10^{5}	1.6×10^{6}
59	0.016	0.229	2.017	0.053	8.8	38.3	3.3	87.3	7.4	2.4×10^{8}	2.2×10^{8}	1.4×10^{5}	2.6×10^{5}	1.7×10^{6}
64	0.022	0.215	1.900	0.052	8.9	36.7	3.1	83.8	8.9	2.6×10^{8}	2.1×10^{8}	3.4×10^{4}	5.9×10^{4}	1.1×10^{6}

Supplementary Table S2. Summary of standard curves for the quantification of archaea, bacteria, cyanobacteria, and plastid.

Target	Type	Template	Calibration equation	Amplification efficiency[a]	Quantification range
Archaea	SYBR	plasmid	y = -3.4377x + 37.294<br>R^2 = 0.9923	95%	1.1×10^1<br>~ 1.1×10^6
Bacteria	SYBR	plasmid	Y = -3.1311 x + 33.684<br>R^2 = 0.9941	109%	1.7×10^1<br>~ 1.7×10^6
Cyanobacteria	SYBR	plasmid	Y = -2.9949 x + 36.666<br>R^2 = 0.9988	116%	4.0×10^1<br>~ 4.0×10^6
Plastid	SYBR	plasmid	Y = -2.9507 x + 34.428<br>R^2 = 0.9962	118%	1.0×10^1<br>~ 1.0×10^6

[a]Amplification efficiency = $(10^{(-1/\text{slope})} - 1) \times 100\%$.

Supplementary Fig. S1. (A-C) *Cyclotella* spp., (D-F) *Aulacoseira granulata*, (G) *Aulacoseira ambigua*, (H-I) *Aulacoseira muzzanensis*, (J) *Synedra ulna*, (K-L) *Fragilaria crotonensis*, and (M-N) *Diatoma vulgare.* Scale bars = 10 µm.

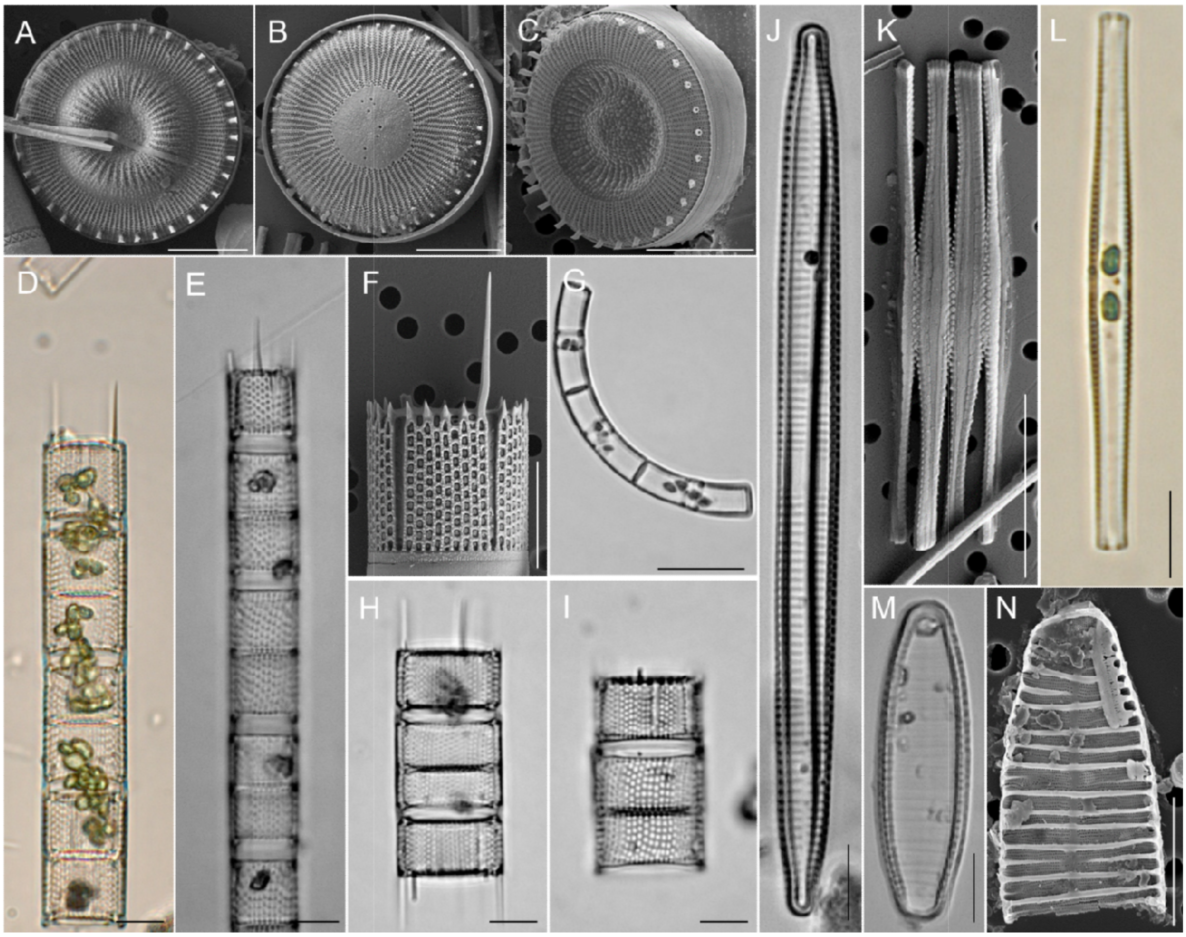

Supplementary Fig. S2. Percentage of sequence reads assigned to each class using an Eztaxon-e database. Percentage reads are classified on the x-axis, and sample number is shown on the y-axis.

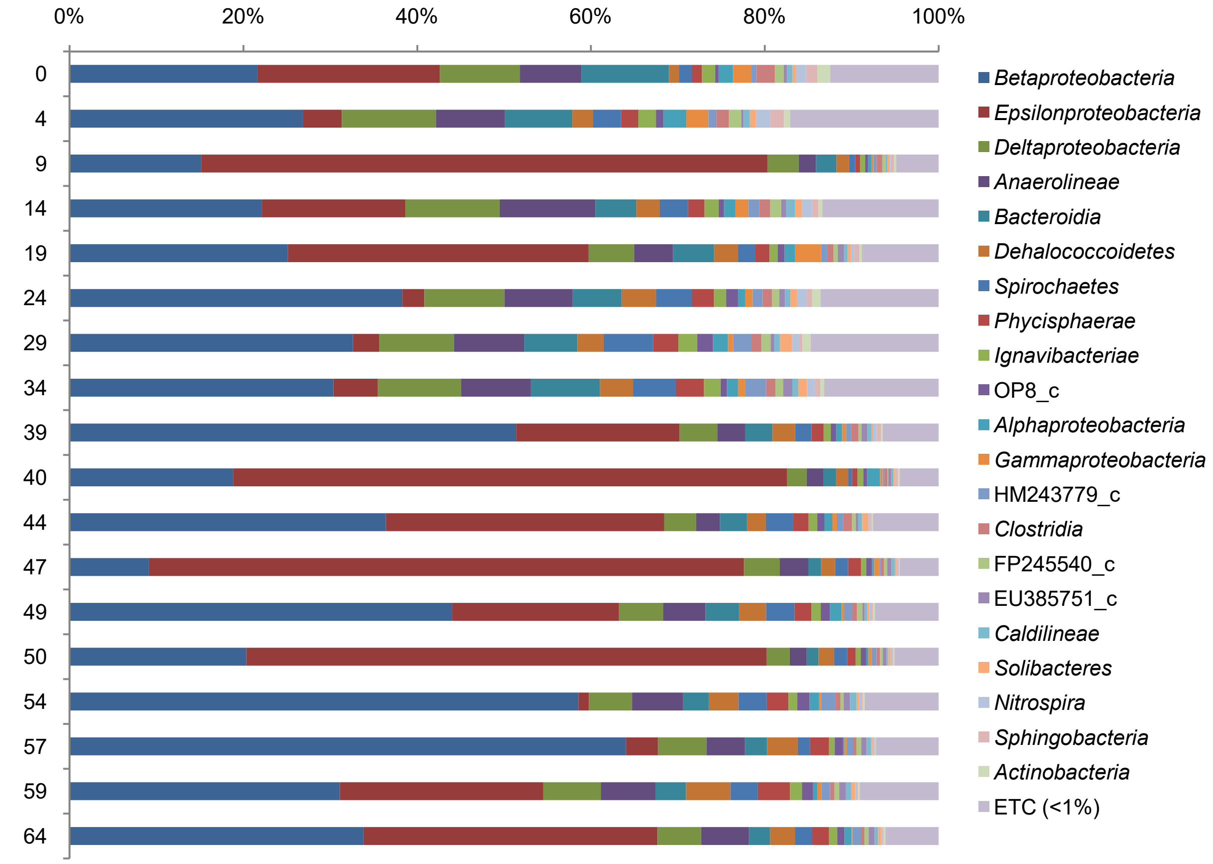

Supplementary Fig. S3. Percentage of sequence reads of the three most abundant genera using an Eztaxon-e database. Percentage reads are classified on the x-axis, and sample number is shown on the y-axis.

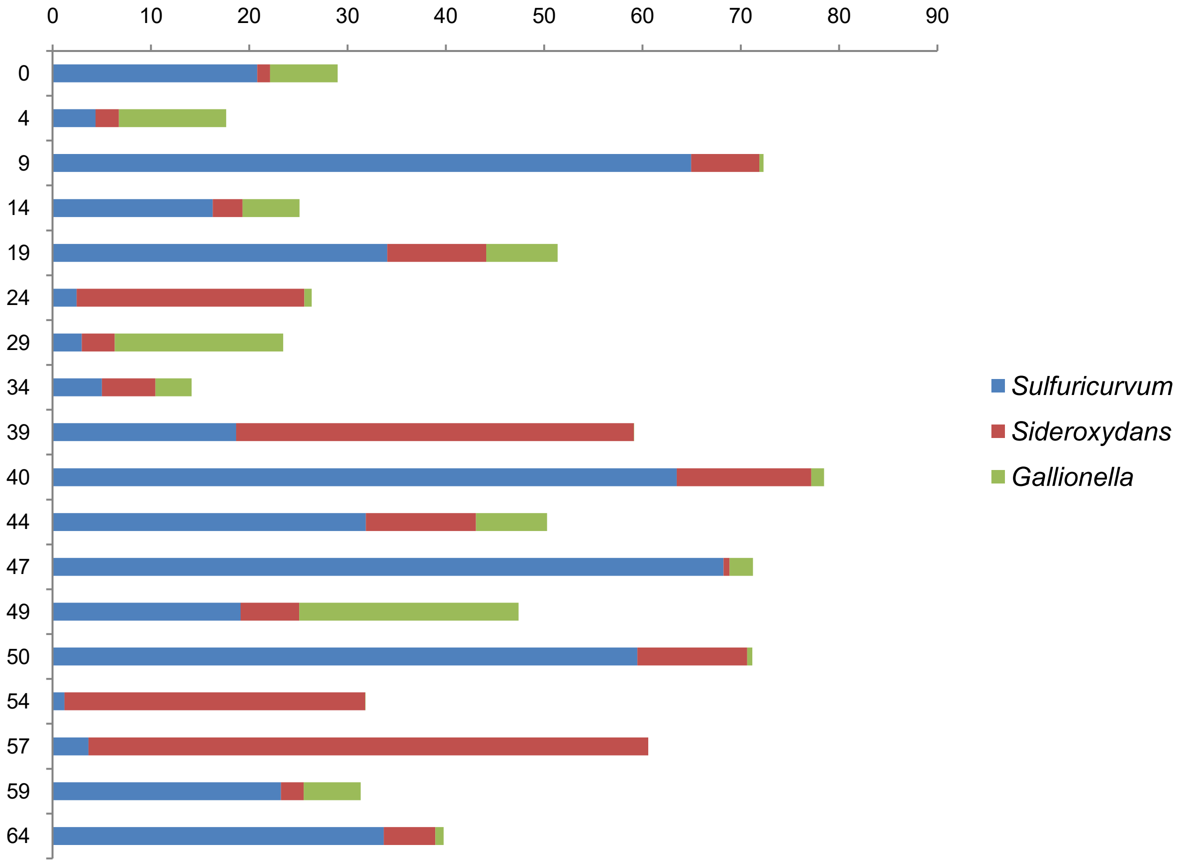

Supplementary Fig. S4. Percentage of sequence reads assigned to each class using an Eztaxon-e database. Sample number is shown on the x-axis, and percentage reads are classified on the y-axis.

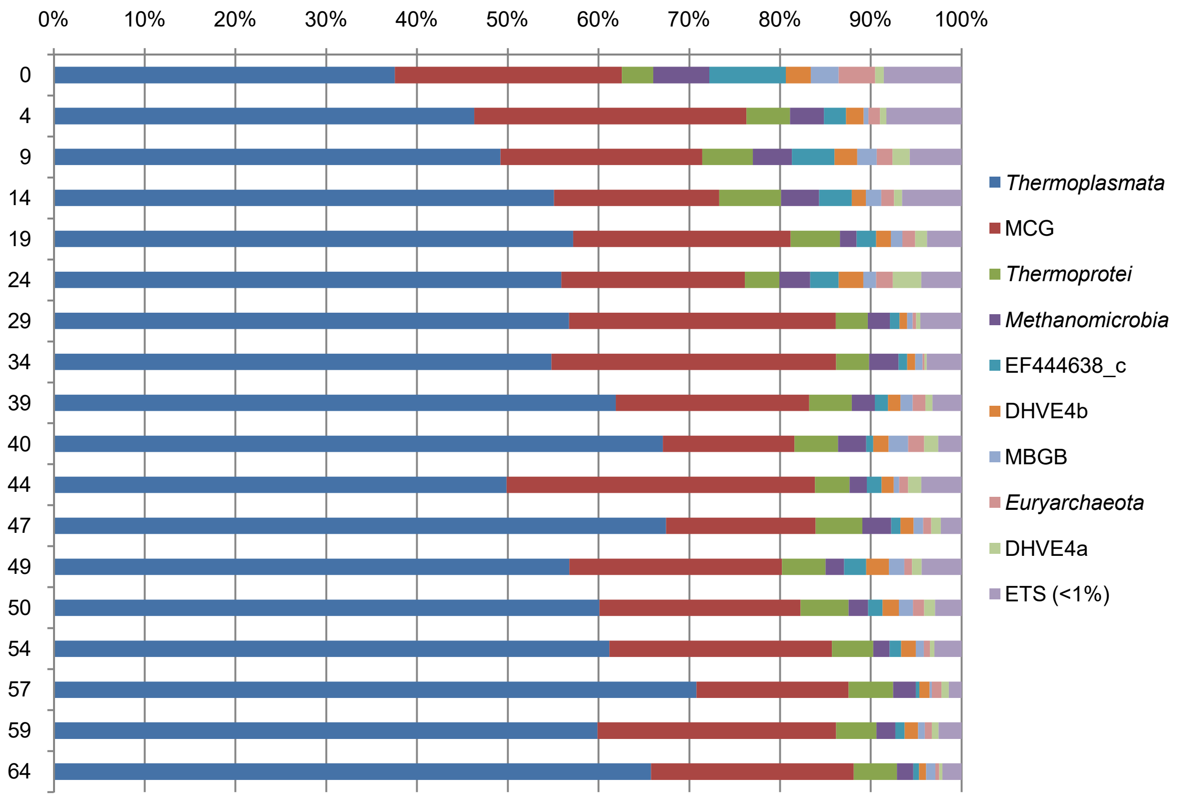

Supplementary Fig. S5. Covariance between the relative abundance of MCG and the context data. The depth distribution of the relative abundance of MCG in all horizon samples strongly covaries with the C/S and C/N ratios in the sediment samples.

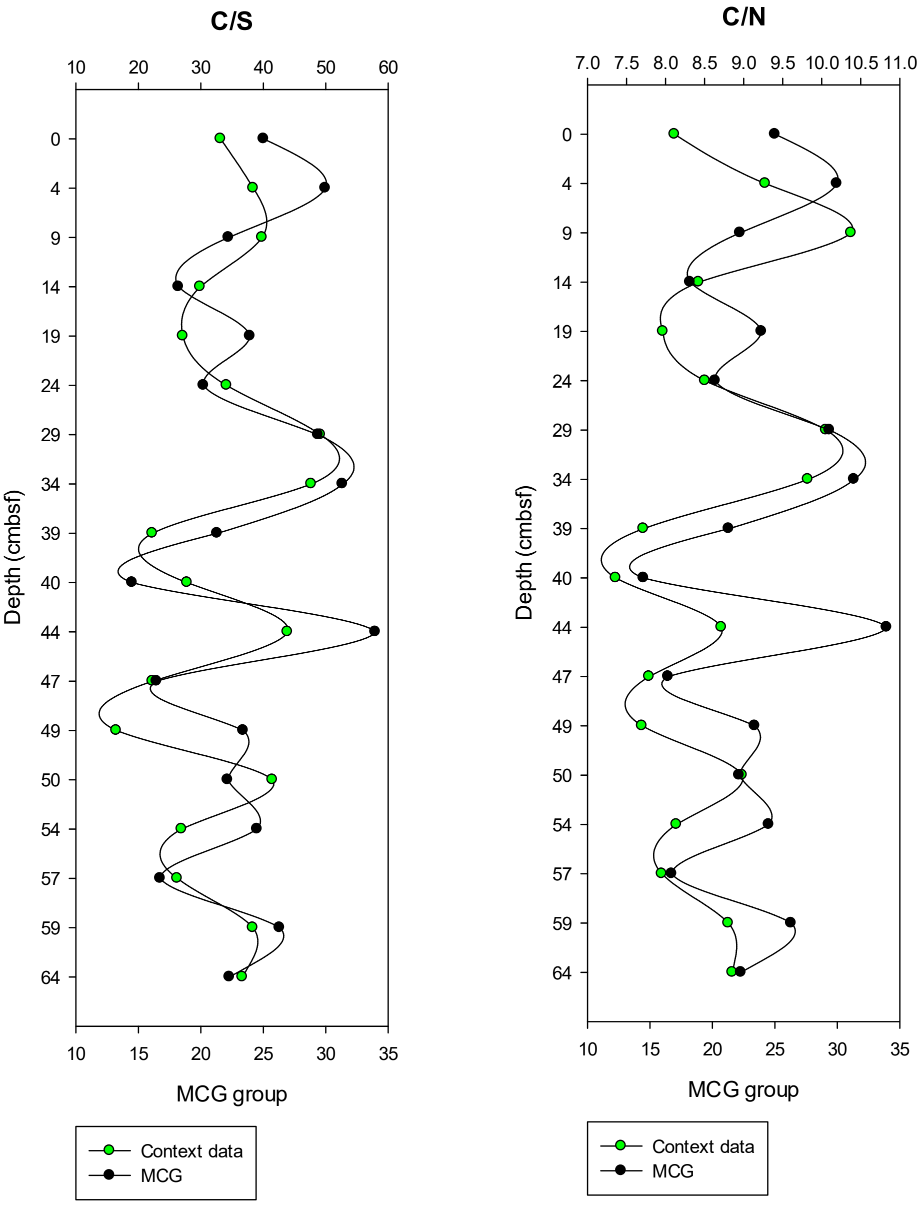

Supplementary Fig. S6. Heat map showing the percent relative abundance of the five most abundant phylotypes at different depths in the sample core with a color legend and a scale provided.

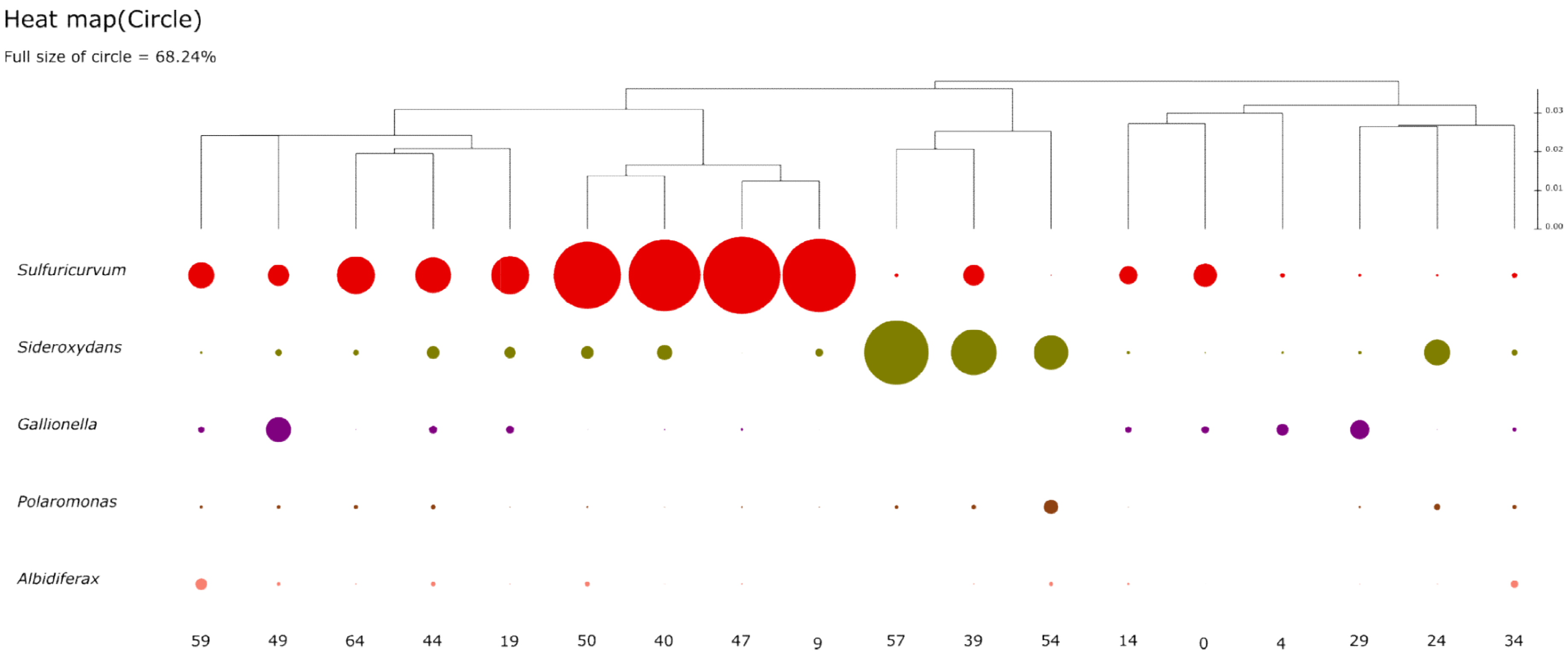

Supplementary Fig. S7. Seasonal variations of temperature, dissolved oxygen and chlorophyll*a* in the freshwaters of Daechung Reservoir.

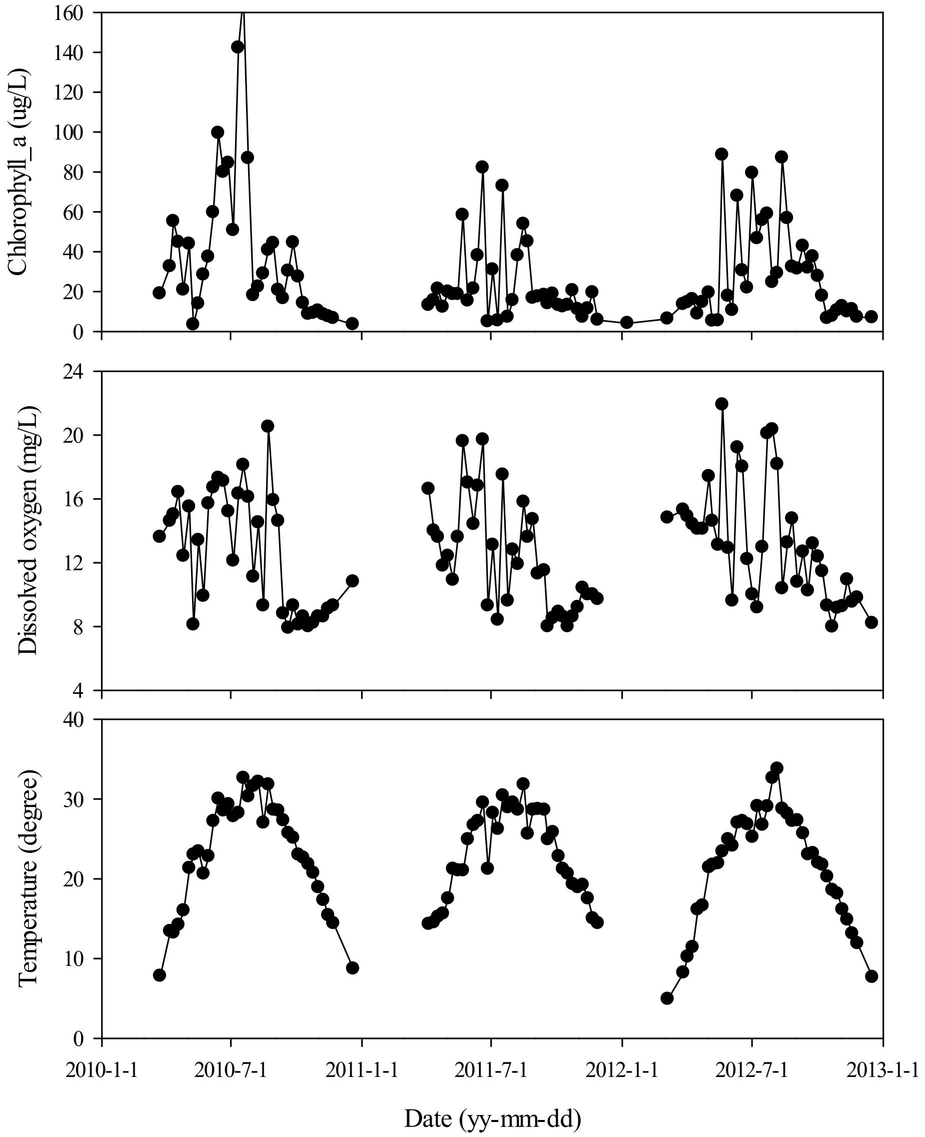

References

1. Hur, M. *et. al*. Effect of genetically modified poplars on soil microbial communities during the phytoremediation of waste mine tailings. *Appl Environ Microbiol* **77**: 7611-7619 (2011).

2. Eddy, S.R. Accelerated profile HMM searches. *PLoS Comput Biol* **7**: e1002195 (2011).

3. Kim, O.S. *et. al*. Introducing EzTaxon-e: a prokaryotic 16S rRNA gene sequence database with phylotypes that represent uncultured species. *Int J Syst Evol Microbiol* **62**: 716-721 (2012).

4. Edgar, R.C., Haas, B.J., Clemente, J.C., Quince, C. & Knight, R. UCHIME improves sensitivity and speed of chimera detection. *Bioinformatics* **27**: 2194-2200 (2011).

5. Schloss, P.D. *et. al*. Introducing mothur: open-source, platform-independent, community-supported software for describing and comparing microbial communities. *Appl Environ Microbiol* **75**: 7537-7541 (2009).

6. Hamady, M., Lozupone, C. & Knight, R. Fast UniFrac: facilitating high-throughput phylogenetic analyses of microbial communities including analysis of pyrosequencing and PhyloChip data. *ISME J* **4**: 17-27 (2010).
